# High-Temperature Synthesis of Ferromagnetic Eu_3_Ta_3_(O,N)_9_ with a Triple Perovskite Structure

**DOI:** 10.1021/acs.inorgchem.3c02691

**Published:** 2023-10-12

**Authors:** Jhonatan R. Guarín, Carlos Frontera, Judith Oró-Solé, Jaume Gàzquez, Clemens Ritter, Josep Fontcuberta, Amparo Fuertes

**Affiliations:** † Institut de Ciència de Materials de Barcelona (ICMAB-CSIC), Campus UAB, 08193 Bellaterra, Spain; ‡ Institut Laue-Langevin, 71 Av. de Martyrs, Grenoble 38000, France

## Abstract

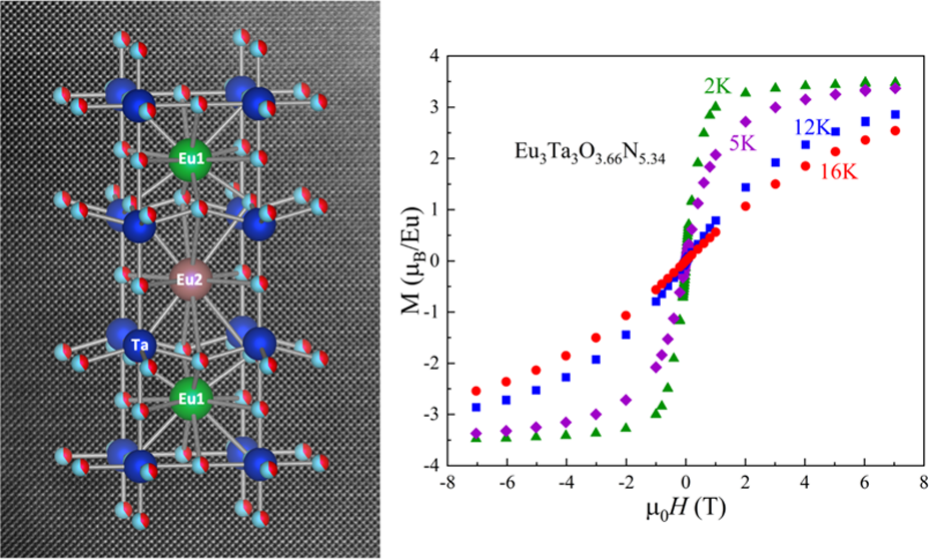

Europium tantalum perovskite oxynitrides were prepared by a new high-temperature solid-state synthesis under N_2_ or N_2_/H_2_ gas. The nitrogen stoichiometry was tuned from 0.63 to 1.78 atoms per Eu or Ta atom, starting with appropriate N/O ratios in the mixture of the reactants Eu_2_O_3_, EuN and Ta_3_N_5_, or Eu_2_O_3_ and TaON, which was treated at 1200 °C for 3 h. Two phases were isolated with compositions EuTaO_2.37_N_0.63_ and Eu_3_Ta_3_O_3.66_N_5.34_, showing different crystal structures and magnetic properties. Electron diffraction and Rietveld refinement of synchrotron radiation X-ray diffraction indicated that EuTaO_2.37_N_0.63_ is a simple perovskite with cubic *Pm*3̅*m* structure and cell parameter *a* = 4.02043(1) Å, whereas the new compound Eu_3_Ta_3_O_3.66_N_5.34_ is the first example of a triple perovskite oxynitride and shows space group *P*4/*mmm* with crystal parameters *a* = 3.99610(2), *c* = 11.96238(9) Å. The tripling of the c-axis in this phase is a consequence of the partial ordering of europium atoms with different charges in two A sites of the perovskite structure with relative ratio 2:1, where the formal oxidation states +3 and +2 are respectively dominant. Magnetic data provide evidence of ferromagnetic ordering developing at low temperatures in both oxynitrides, with saturation magnetization of about 6 μ_B_ and 3 μ_B_ per Eu ion for EuTaO_2.37_N_0.63_ and the triple perovskite Eu_3_Ta_3_O_3.66_N_5.34_ respectively, and corresponding Curie temperatures of about 7 and 3 K, which is in agreement with the lower proportion of Eu^2+^ in the latter compound.

## Abstract 2

Europium tantalum perovskite oxynitrides are prepared by a new solid-state synthesis approach under N_2_ or N_2_/H_2_ at 1200 °C starting with metal nitrides and oxides. N/O tuning in the mixture of reactants allows to obtain the first triple perovskite oxynitride Eu_3_Ta_3_O_3.66_N_5.34_ where europium cations order in two A′ and A″ sites with ratio 2:1 occupied preferentially by Eu^3+^ and Eu^2+^, respectively. The new compound is ferromagnetic with *T*_c_ ≈ 3 K.

## Introduction

Perovskite oxynitrides AB(O,N)_3_ (A = alkaline earth or rare earth metal; B = transition) are important materials with electronic properties and photocatalytic activity of relevance in several reactions.^1^ The majority of reported compounds show crystal structures derived from the *Pm*3̅*m* aristotype, frequently showing lower symmetry space groups resulting from a combination of octahedral tilting and anion order.^2−4^ Perovskite oxynitrides with more complex structures have been also reported, although the number of compounds is restricted to a few examples. Double perovskites with B-site order A_2_B′B″(O,N)_6_ have been reported for three compounds with the pairs of cations B′/B″= Fe^3+^/W^6+^,^5^ Fe^3+^/Mo^6+^,^6^ and Mn^2+^/Ta^5+^.^7^ Layered Ruddlesden–Popper^8,9^ (A_*n*+1_B*_n_*O_3*n*+1_) phases with *n* = 1 (A_2_B(O,N)_4_) and *n* = 2 (A_3_B_2_(O,N)_7_) have been reported for six compounds containing Nb,^10^ Ta,^11−13^ or Al,^14^ and Dion-Jacobson structures^15,16^ A′[A″_*n*–1_B*_n_*(O,N)_3*n*+1_] have been found for A′ = alkaline metal, A″ = La, Ca, and B = Ta, Nb.^17,18^ In the group of hexagonal perovskite oxynitrides, the only known compound is BaWON_2_ that shows the 6H polytype.^19^

Europium perovskite oxynitrides EuB(O,N)_3_ (B = Ti, Nb, W, Ta) have been investigated for their electrical and magnetic properties, which are affected by the N/O balance that tunes the formal valence state of Eu and the B cations: Eu^2+^ to Eu^3+^, and those of transition metals Nb^4+^, Nb^5+^, W^5+^, and W^6+^. For instance, EuNbO_2+*x*_N_1–*x*_ (*x* ≤ 0.14)^20^ and EuWO_1+*x*_N_2–*x*_ (−0.16 ≤ *x* ≤ 0.46)^21^ show ferromagnetic ordering of Eu^2+^*S* = 7/2 spins below 5.2 and 12 K, respectively. In EuWO_1+*x*_N_2–*x*_, the electrical conductivity changes with the N/O ratio, and in both Nb and W compounds colossal magnetoresistance emerges below the Curie temperature, arising from the coupling between the localized Eu^2+^ spins and the transition metal (4,5)d carriers. EuTiO_3–*x*–*y*_N_*x*_ with nitrogen contents up to *x* = 1 has been also reported,^22^ with the N/O ratio and the anion vacancies tuning the europium oxidation state and the electronic properties.

The europium tantalum oxynitride perovskite EuTaO_2_N was first prepared by Marchand et al. by the treatment of EuTaO_4_ under NH_3_ at 950 °C.^23^ More recently, we prepared this oxynitride in similar conditions with a small nitrogen nonstoichiometry EuTaO_2–*x*_N_1+*x*_ (0 ≤ *x* ≤ 0.2), formally involving the presence of a low proportion of Eu^3+^ for *x* > 0, and ferromagnetism was observed below *T*_c_ = 5.1 K for a sample with *x* = 0.05. The laboratory X-ray diffraction pattern of the EuTaO_2_N sample could be indexed in a cubic cell with *a* = 4.0217(2) Å, but synchrotron X-ray diffraction indicated a small tetragonal distortion with *a* = 4.02054(2), *c* = 4.03079(4) Å.^20^ Electron diffraction of EuTaO_2_N, EuNbO_2_N, and EuWO_2_N shows a √2*a*_0_ × √2*a*_0_ × 2*a*_0_ superstructure (where *a*_0_ is the parameter of the perovskite cubic subcell) that was ascribed to octahedral tilting.^20,21^ Disordered B-site perovskites with compositions EuTi_0.5_W_0.5_O_3–*x*_N_*x*_ and nitrogen contents between 0.87 and 1.63 show ferromagnetic and antiferromagnetic exchange interactions between the Eu^2+^ cations, and the magnetic properties are tuned by the equilibrium Eu^2+^ + W^6+^ ↔ Eu^3+^ + W^5+^ which is shifted to the right for larger *x* values.^24^

The synthesis of all previously reported europium perovskite oxynitrides has been performed by ammonolysis of precursors at temperatures below 1000 °C. In this paper, we report the study of the crystal structure and magnetic properties of EuTaO_3–*x*_N_*x*_ compounds with a large range of N/O contents, prepared by a new synthetic approach that uses solid-state reactions between metal nitrides and oxides under N_2_ or N_2_/H_2_ gas at relatively high temperature (1200 °C). Two phases have been isolated with EuTaO_2.37_N_0.63_ and Eu_3_Ta_3_O_3.66_N_5.34_ stoichiometries showing different perovskite structures, as determined from synchrotron X-ray diffraction and electron diffraction. EuTaO_2.37_N_0.63_ is a simple Eu^2+^ cubic perovskite similar to previously reported EuTaO_2_N but with a large proportion (37%) of reduced Ta^4+^. The compound Eu_3_Ta_3_O_3.66_N_5.34_ represents the first example of an oxynitride with a triple perovskite structure, which is a consequence of the partial ordering of Eu^2+^ and Eu^3+^ ions in the A sites. The magnetic data are found to be fully consistent with this finding, with both oxynitrides displaying a ferromagnetic ordering at low temperatures, with Curie temperatures of about 7 K for EuTaO_2.37_N_0.63_ and somewhat lower (≈3 K) for Eu_3_Ta_3_O_3.66_N_5.34_ due to dilution effects of magnetic interactions in the latter compound.

## Experimental Methods

### Synthesis and Chemical Characterization

Samples of 130 mg with compositions EuTaO_3–*x*_N_*x*_ (0.63 ≤ *x* ≤ 1.78) were prepared using the reactants Eu_2_O_3_ (Sigma-Aldrich 99.9%), EuN (Materion, 99.9%), TaON, and Ta_3_N_5_. The N/O ratio in the initial mixture was the most determining factor in the final nitrogen content of the sample. This was changed by varying the proportion of the reactants while keeping constant the Eu/Ta ratio of 1:1. Eu_2_O_3_ was treated at 900 °C under a dynamic vacuum of 10^–3^ Torr for dehydration. Ta_3_N_5_ was prepared by the treatment of Ta_2_O_5_ (Sigma-Aldrich, 99.99%) at 850 °C under NH_3_(*g*) (Carburos Metálicos, 99.9%), at a flow rate of 600 cm^3^/min, using several cycles of 15 h with intermediate regrinding. TaON was prepared by the treatment of Ta_2_O_5_ at the same temperature under NH_3_(g) at a flow rate of 40 cm^3^/min, using two cycles of 3 h with intermediate regrinding.^25^ Handling of the reactants, mixing, and pelletizing were done in a glovebox under a recirculating Ar atmosphere. The pellets were placed in a molybdenum crucible covered by zirconium foil, which was also used for oxygen and water scavenging in a second crucible placed close to the sample in the furnace tube (Al_2_O_3_, Alsint 99.7%). The samples were heated at 300 °C/h up to 1200 °C under flowing N_2_ (Air Liquide, 99.9999%) or N_2_/H_2_ (95%/5% v/v, Air Liquide, 99.9999%), treated for 3 h at 1200 °C, and cooled down to room temperature.

Nitrogen contents were determined by combustion analysis performed in a ThermoFisher Scientific instrument, heating the samples in oxygen up to 1060 °C and using MgO, WO_3_, and Sn as additives and atropine as a reference standard. EDX analyses of cation contents were performed in a FEI Quanta 200 FEG microscope equipped with an EDAX detector with an energy resolution of 132 eV. The analyses were performed on 10–15 crystallites for each sample.

### Structural Characterization

Laboratory X-ray powder diffraction data were acquired on a Panalytical X’Pert Pro MPD diffractometer using Cu Kα radiation (λ = 1.5418 Å). High-resolution synchrotron X-ray powder diffraction data were measured at room temperature from capillary samples (0.3 mm diameter) in the angular range 2.0° ≤ 2θ ≤ 56.9° at the MSPD beamline^26^ of the ALBA Synchrotron (Cerdanyola del Vallès, Spain). A short wavelength of 0.45872 Å calibrated with Si NIST was selected by using a double Si(111) and Si(220) crystal monochromator. Background refinement was performed by linear interpolation, and data were corrected from absorption.

Neutron powder diffraction data were collected for 12 h at room temperature on the high-intensity D20 diffractometer at the Institut Laue-Langevin (ILL), France. In order to reduce the absorption from Eu, a double wall vanadium can was used as a sample holder, and a short wavelength of 1.37 Å at the high 118° take-off angle giving high resolution was chosen. The step scanning mode where the detector was moved in 61 steps of 0.05° was chosen in order to compensate for the nonperfect calibration of the more than 3000 detector cells. Rietveld analysis was carried out using the program Fullprof.^27^

Electron diffraction micrographs were obtained in a JEOL 1210 transmission electron microscope operating at 120 kV using a side entry double tilt ±60°/±30° specimen holder. The samples were prepared by dispersing the powders in hexane and depositing a droplet of the suspension on a copper grid coated with a holey carbon film. The local microstructure of the samples was analyzed by means of scanning transmission electron microscopy (STEM) on a ThermoFisher Spectra 300 operated at 300 kV. The high-angle annular dark field detector allows for recording incoherent *Z*-contrast images, in which the contrast of an atomic column is approximately proportional to the square of the average atomic number (*Z*). Accordingly, it is possible to distinguish between Ta and Eu. The experiments were performed in the Joint Electron Microscopy Center at ALBA (Cerdanyola del Vallès, Spain).

### Magnetic Measurements

Magnetic measurements were performed at fields of 25 Oe and 10 kOe between 2 and 300 K using a Quantum Design SQUID magnetometer. Magnetization-field loops were measured between −70 and +70 kOe between 2 and 16 K.

## Results and Discussion

### Synthesis and Structural Study of EuTaO_2.37_N_0.63_ and Eu_3_Ta_3_O_3.66_N_5.34_

The synthesis of europium tantalum perovskite oxynitride samples is performed at high temperatures under N_2_/H_2_ (95%/5% v/v) or N_2_ gas, using one of the following solid-state reactions with one single treatment of 3 h at 1200 °C
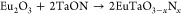
1
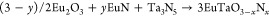
2

The reaction used, the proportions of the reactants, the selected gas, and the maximum synthesis temperature determined the average nitrogen content of the sample per Eu or Ta mol, which was tuned from *x* = 0.63 to 1.78, and the phase composition. We have recently reported a similar synthetic approach for the preparation of LaTaON_2_ and slightly nitrogen-deficient LaTaO_1.12_N_1.88_ that we investigated for their dielectric properties.^28^ Both compounds were prepared either from LaN and TaON or from La_2_O_3_, LaN, and Ta_3_N_5_ at 1500 °C. In the EuTaO_3–*x*_N_*x*_ samples, the syntheses performed at 1500 °C led to partial decomposition into TaN and Eu_3_TaO_6_ phases; hence, a lower temperature of 1200 °C was selected.

**Figure 1:**
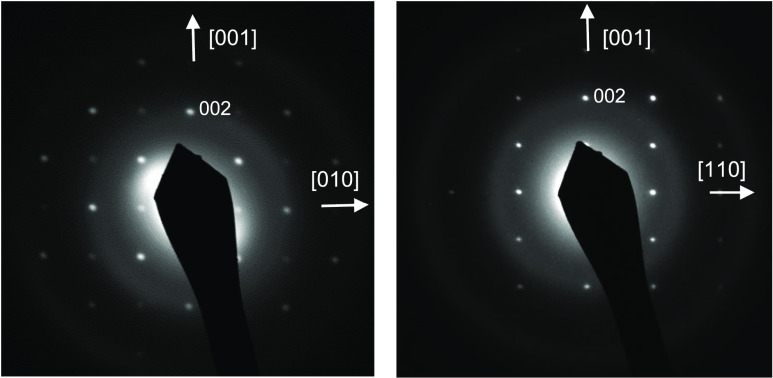
Electron diffraction patterns along the [100] and [1̅10] axes of EuTaO_2.37_N_0.63_.

**Figure 2:**
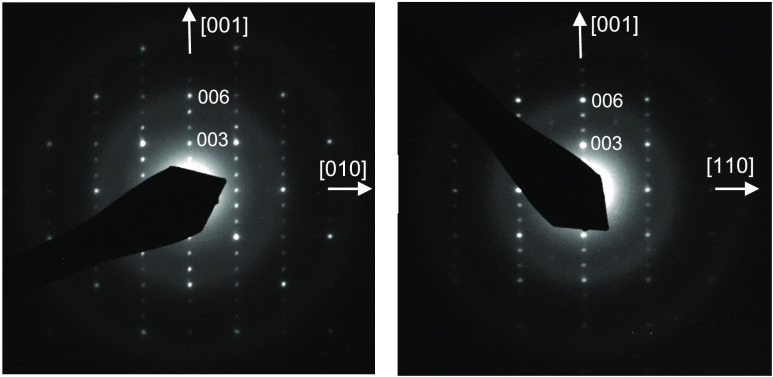
Electron diffraction patterns along the [100] and [1̅10] axes of Eu_3_Ta_3_O_3.66_N_5.34_.

Two different perovskite phases were isolated, with stoichiometries EuTaO_2.37_N_0.63_ (phase I) and Eu_3_Ta_3_O_3.66_N_5.34_ (phase II) that showed black and brown colors, respectively. The Eu/Ta ratios using EDX analysis were 0.93(6) for phase I and 0.94(10) for phase II, whereas the errors in the nitrogen contents obtained by combustion analysis were ±0.03 in both cases. The oxygen stoichiometry was calculated by difference, assuming that the total anion content was, respectively, three and six atoms per formula for phases I and II. EuTaO_2.37_N_0.63_ was prepared using reaction 1 in N_2_/H_2_ (95%/5% v/v) gas, which favored the reduction of the cations. The observed nitrogen content in this sample involved a decrease in the N/O ratio with respect to the initial composition (from 0.4 to 0.27). Considering the charge compensation, this stoichiometry is consistent with the presence of reduced Ta and Eu cations with the formal plausible composition Eu^2+^(Ta_0.37_^4+^Ta_0.63_^5+^)O_2.37_N_0.63_. The existence of 100% of europium in the divalent state agrees with the observed effective magnetic moment of this compound (see below), whereas the +4 oxidation state of tantalum has been suggested in other perovskite oxynitrides coexisting with the more stable Ta^5+^ cation.^28−30^ The electron diffraction patterns of EuTaO_2.37_N_0.63_ indicated a cubic perovskite cell of *a* ≃ 4.0 Å with the space group of aristotype *Pm*3̅*m* (Figure 1). This result differs from our previously reported electron diffraction study of EuTaO_2_N prepared by ammonolysis, which showed additional reflections indicative of a tilted *I2*/*m* superstructure with *a*, *b*= √2 *a*_0_ and *c* = 2 *a*_0_.^2,20^ The perovskite Eu_3_Ta_3_O_3.66_N_5.34_ (phase II) was prepared with reaction 2 at the same temperature than EuTaO_2.37_N_0.63_, under N_2_ with *y* = 1.8 (initial ratio N/O of 3.78). The electron diffraction patterns of this phase showed a 3 × *a*_0_ superstructure along one of the axes of the perovskite subcell (Figure 2). The reconstruction of the reciprocal lattice leads to a tetragonal cell with parameters *a ≃* 4.04, *c* ≃ 12.08 Å and reflection conditions compatible with the space group *P*4/*mmm*. The study by electron diffraction of samples prepared using reaction 2 but starting with N/O ratios below 3.78 invariably led to the observation of a coexistence of two phases: the compound II and an additional perovskite phase, with symmetry *I2*/*m* and *a*, *b*= √2*a*_0_ and c= 2*a*_0_, which is the same as previously reported for our EuTaO_2_N sample prepared by ammonolysis.^20^ The biphasic nature of these samples was also clearly observed in the laboratory X-ray diffraction patterns.

**Figure 3:**
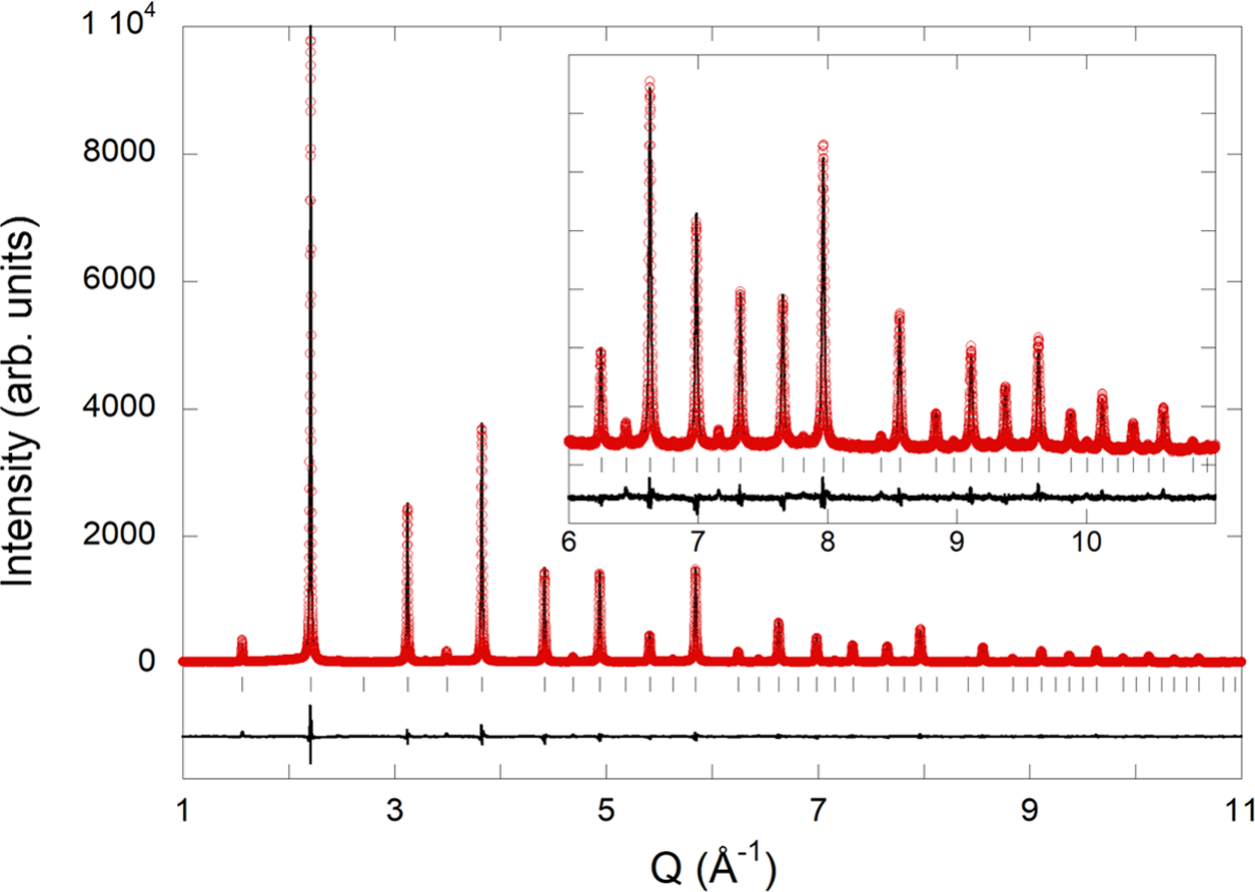
Rietveld fit to synchrotron X-ray powder diffraction pattern of EuTaO_2.37_N_0.63_ performed in space group *Pm*3̅*m* with *a* = 4.02044(1) Å. The inset shows the high *Q* region enlarged (where *Q* = (4π sin θ)/λ). Agreement factors: *R*_Bragg_ = 3.73%, *R*_p_ = 4.76%, *R*_wp_ = 6.13%, χ^2^ = 4.03.

Rietveld refinement of synchrotron X-ray diffraction data of EuTaO_2.37_N_0.63_ (Figure 3) was performed in the space group *Pm*3̅*m* with *a* = 4.02044(1) Å (*V* = 64.986 Å^3^), using a common temperature factor for all atoms *B* = 0.818(2) Å^2^. The observed bond distances are *d*(Eu–O,N) = 2.843 Å and *d*(Ta–O,N) = 2.010 Å. The cell parameter is close to that shown by EuTaO_2_N (*a* = 4.0217(2) Å) prepared by ammonolysis,^20^ indicating that the decrease in *a* caused by the lower nitrogen content (*r*(N^3–^) = 1.46 Å vs *r*(O^2–^)=1.38 Å both for CN = IV) is compensated by the increase induced by the presence of Ta^4+^ (*r*(Ta^5+^)= 0.64 Å, *r*(Ta^4+^)= 0.68 Å, both for CN = VI).^31^

**Figure 4:**
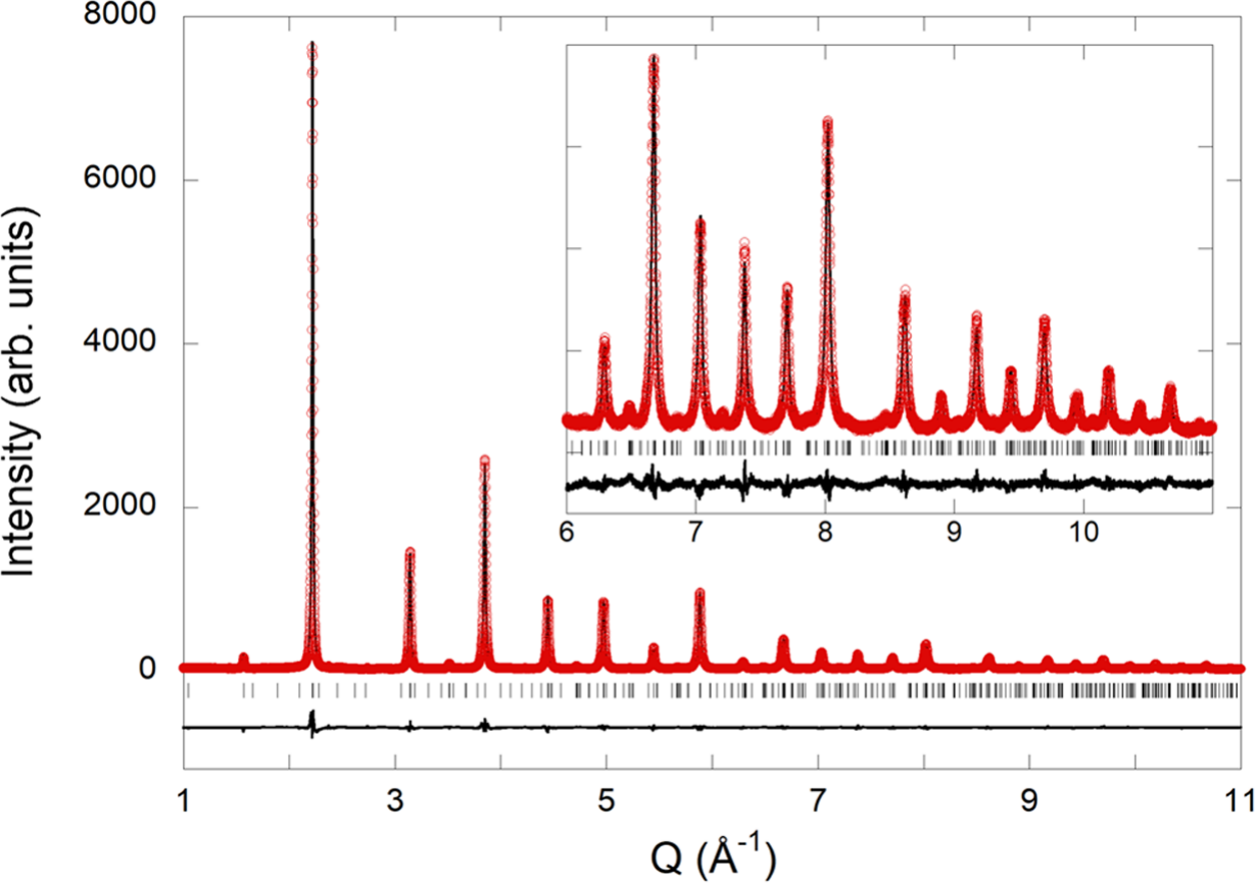
Rietveld fit to synchrotron X-ray powder diffraction pattern of Eu_3_Ta_3_O_3.66_N_5.34_ performed in the *P*4/*mmm* space group with parameters *a* = 3.99610(2), *c* = 11.96238(9) Å. The inset shows the high *Q* region enlarged.

**Figure 5:**
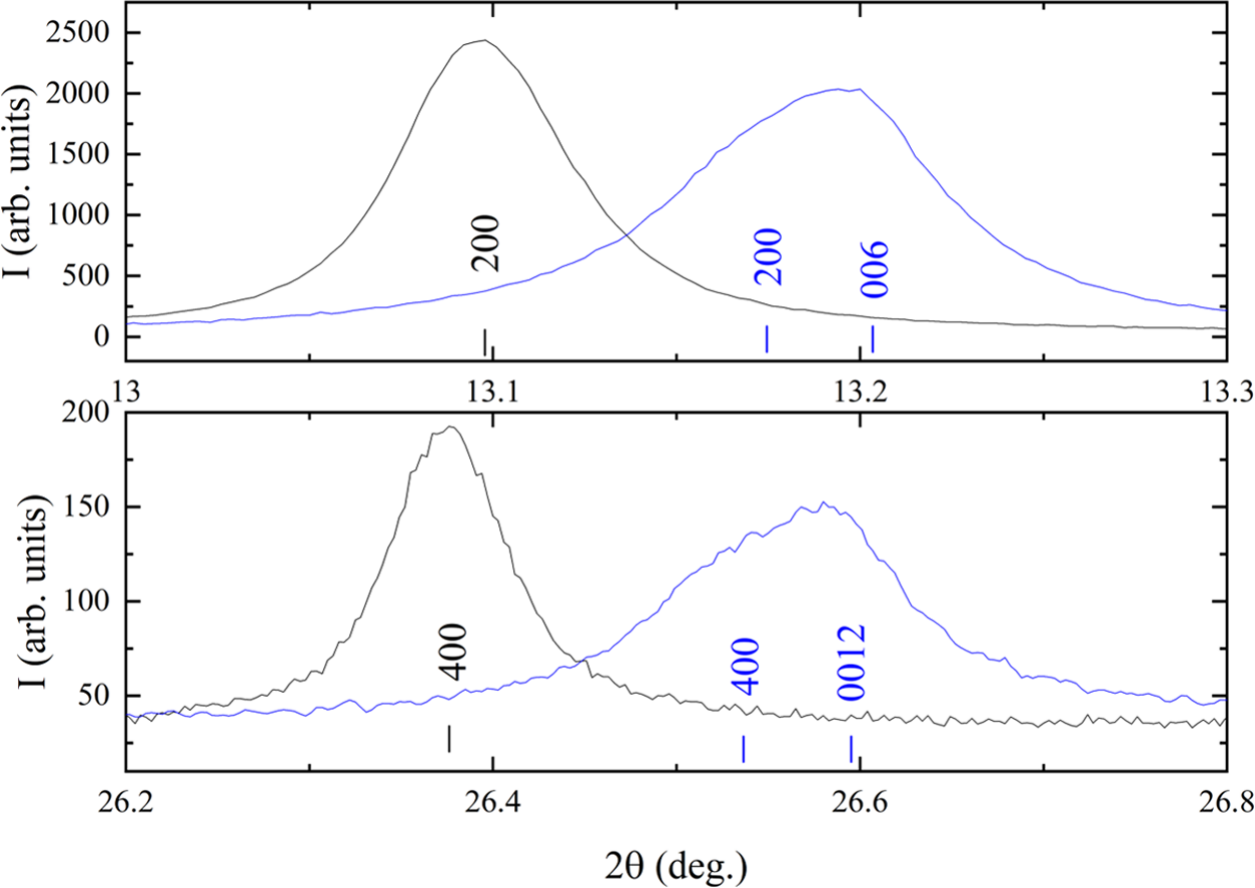
Synchrotron X-ray powder diffraction profiles in two 2θ regions of EuTaO_2.37_N_0.63_ and Eu_3_Ta_3_O_3.66_N_5.34_ are depicted in black and blue colors, respectively.

**Figure 6:**
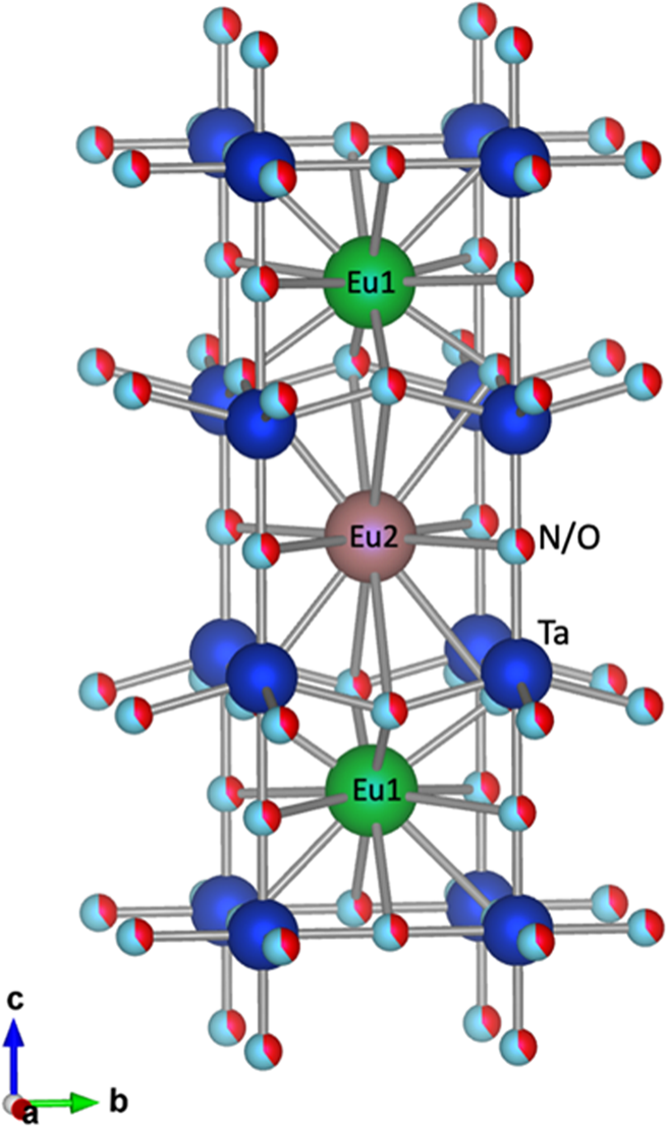
Structural model of the triple perovskite Eu_3_Ta_3_O_3.66_N_5.34_.

**Figure 7:**
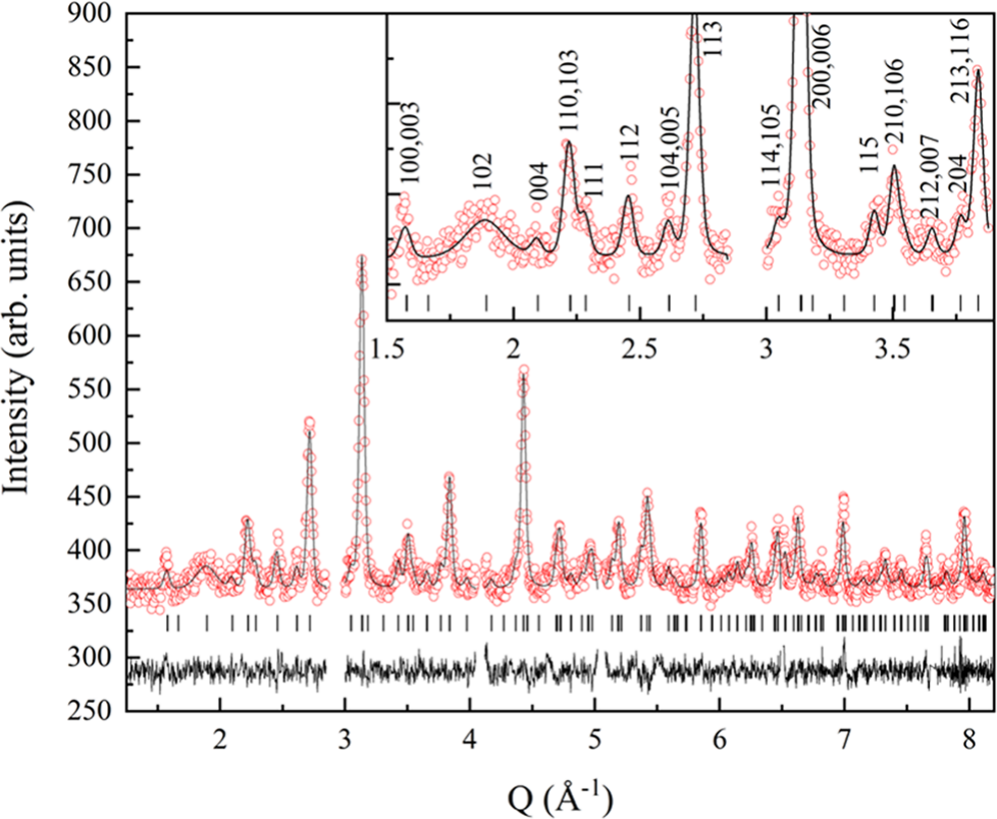
Le Bail fit of neutron diffraction data for phase II (λ = 1.37 Å) indexed (inset) in a *P*4/*mmm* unit cell with parameters *a* = 4.0265(2) and *c* = 12.0949(12) Å. Excluded regions correspond to peaks from the V sample holder.

The synchrotron X-ray powder diffraction of Eu_3_Ta_3_O_3.66_N_5.34_ (Figure 4) did not show clearly visible superstructure peaks of the triple cell, but a tetragonal splitting is observed for several reflections even at low angles, as well as significant broadening in all peaks with respect to the cubic compound EuTaO_2.37_N_0.63_ (see Figure 5). A Rietveld refinement in a tetragonal subcell with parameters *a* = 3.98994(2), *c* = 3.9968(5) Å and space group *P*4/*mmm* was performed with one position for Eu and Ta at sites 1d and 1a respectively, and two anion positions at 0, 1/2, 0 *(*2f site) and 0, 0, 1/2 (1b site). This led to poor agreement factors, with *R*_Bragg_ = 8.45%, *R*_wp_ = 7.97%, and χ^2^ = 4.90. In contrast, the refinement performed using a triple perovskite structure model with parameters *a* = 3.99610(2), *c* = 11.96238(9) Å in the space group *P*4/*mmm* and two crystallographically independent sites for both Eu and Ta atoms (Figures 4 and 6 and Table 1) showed significantly improved agreement factors, with *R*_Bragg_ = 5.64%, *R*_wp_ = 7.19%, and χ^2^ = 3.74. For the nitrogen and oxygen atoms, we considered a statistical distribution in the four available sites, because the X-rays do not provide enough contrast between the two anions. In order to investigate the potential anion order, neutron diffraction data were acquired on a 380 mg sample prepared in the same conditions as Eu_3_Ta_3_O_3.66_N_5.34_, that showed close nitrogen content (1.91(3) atoms per perovskite unit), similar electron diffraction patterns, and refined parameters from X-ray diffraction *a* = 3.98919(2), *c* = 12.00107(11) Å. These data clearly showed superstructure peaks that were indexed in the triple perovskite unit cell. However, the large absorption cross-section of europium and the small sample mass strongly limited the quality of the data and prevented the extraction of reliable structural data from the Rietveld refinement. A Le Bail fit performed using the Fullprof program without introducing any structural model returned the refined parameters *a* = 4.0262(2) and *c* = 12.0959(7) Å (Figure 7). The small deviations between the cell parameters obtained by neutron diffraction and X-ray diffraction for this sample are due to differences in the resolution and quality between the two sets of data, caused by the strong Eu absorption in neutron diffraction.

**Table 1:** Summary of the *P*4/*mmm* Model for Eu_3_Ta_3_O_3.66_N_5.34_ Refined against Room Temperature Synchrotron X-ray Powder Diffraction Data Using λ= 0.45872 Å^a^^,^^b^^,^^c^

atom	site	*x*	*y*	*z*	occupancy
Eu1	2h	0.5	0.5	0.1690(2)	1
Eu2	1d	0.5	0.5	0.5	1
Ta1	2g	0	0	0.33464(15)	1
Ta2	1a	0	0	0	1
O1/N1	2g	0	0	0.1522(13)	0.4/0.6
O2/N2	4i	0.5	0	0.2887(6)	0.4/0.6
O3/N3	1b	0	0	0.5	0.4/0.6
O4/N4	2f	0.5	0	0	0.4/0.6

bond	distance (Å)	bond	distance (Å)	bond	distance (Å)
Eu1–O1,N1	2.833(1) × 4	Eu1–O2,N2	2.458(4) × 4	Eu1-(O4,N4)	2.842(2) × 4
Eu2–O2,N2	3.222(6) × 8	Eu2–O3,N3	2.826 × 4		
Ta1–O1,N1	2.182(16) × 2	Ta1–O2,N2	2.072(2) × 2	Ta1–O3,N3	1.978(2) × 2
Ta2–O1,N1	1.821(16) × 2	Ta2–O4,N4	1.998 × 4		

a Cell parameters: *a* = 3.99610(2), *c* = 11.96238(9) Å. *V* = 191.025(2) Å^3^. *R*_Bragg_ = 5.64%, *R*_wp_ = 7.19%, χ^2^ = 3.74.

b Average bond distances (Å): Eu1–O,N 2.711; Eu2–O,N 3.090; Eu–O,N 2.90; Ta1–O,N 2.078; Ta2–O,N 1.939. Bond angles (deg): Ta1-(O2,N2)-Ta1 149.2(3).

c Estimated standard deviations in parentheses are shown once for each independent variable. Isotropic thermal parameters were refined to *B* = 0.657(3) Å^2^ for all sites.

The structural data in Table 1 show that the observed average bond distance around the europium atom at the 1d site (*d*(Eu2–O,N) = 3.090 Å) is significantly larger than for Eu1 at the 2h site (2.711 Å). Considering charge compensation and the analyzed nitrogen stoichiometry of this sample (1.78 per Eu mol), phase II is formally mixed-valence Eu_2.34_^3+^Eu_0.66_^2+^Ta_3_O_3.66_N_5.34_. According to the structural data and the differences in the ionic radii between Eu^2+^ and Eu^3+^ (*r* Eu^3+^(CN IX) = 1.120 Å vs *r* Eu^2+^(CN IX) = 1.30 Å),^31^ the triple perovskite structure is plausibly formed from two ordered A sites A1 and A2 with different charge and ratio 2:1, that show preferred occupancy by Eu^3+^ and Eu^2+^ respectively creating distinct anion environments. A recent example of mixed-valence europium tantalum oxynitride is the *n* = 2 Ruddlesden–Popper compound Eu^2+^Eu_2_^3+^Ta_2_O_3_N_4_ that shows, as Eu_2.34_^3+^Eu_0.66_^2+^Ta_3_O_3.66_N_5.34_, a larger proportion of Eu^3+^ related to Eu^2+^. In Eu^2+^Eu_2_^3+^Ta_2_O_3_N_4_, the Eu^2+^ and Eu^3+^ cations order respectively in the rock-salt and in the perovskite-type positions of the Ruddlesden–Popper structure.^13^ The unit-cell volumes of the two europium tantalum perovskites EuTaO_2.37_N_0.63_ and Eu_3_Ta_3_O_3.66_N_5.34_ are *V*_I_ = 64.986 Å^3^ and *V*_II_ = 191.025(2) Å^3^ respectively, which after normalizing to the cubic perovskite subcell (64.986 and 63.675 Å^3^ respectively) show a decrease with increasing the nitriding degree. This is a consequence of the oxidation of the cations that overcompensates the increase caused by the larger radius of N^3–^ compared to O^2–^.

**Figure 8:**
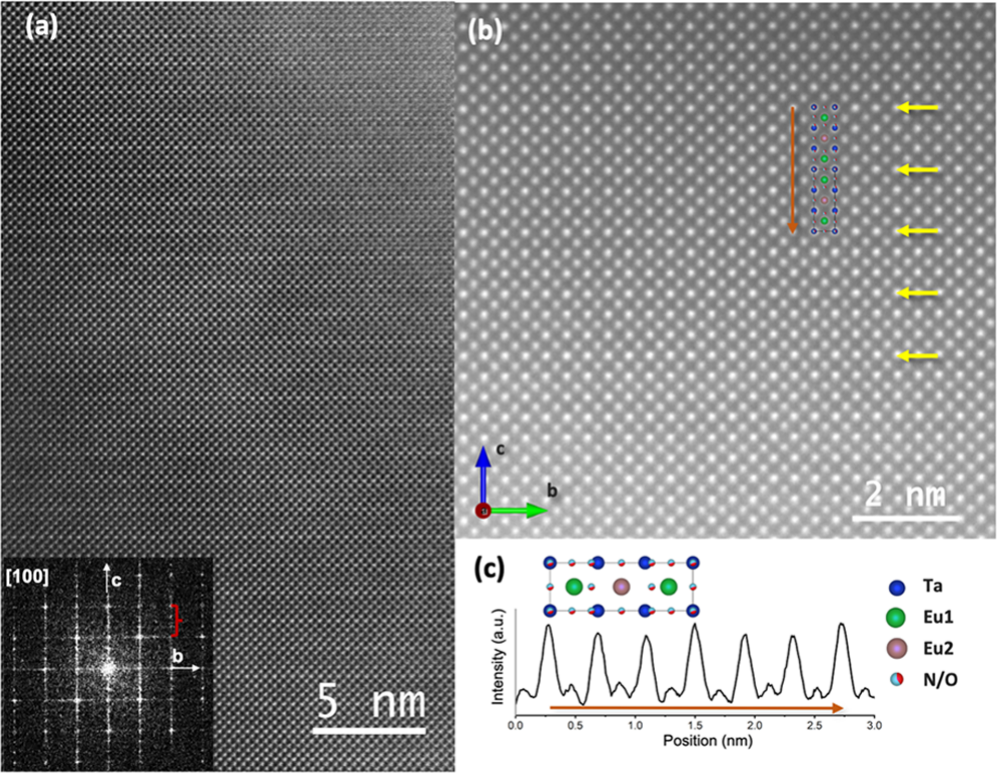
(a) High-resolution *Z*-contrast image of the Eu_3_Ta_3_O_3.66_N_5.34_ triple perovskite compound viewed along the [100] zone axis. The inset shows the Fourier Transform of the *Z*-contrast image, in which the extra Bragg stemming from the superstructure is indicated with a red bracket. (b) Atomic-resolution *Z*-contrast image of Eu_3_Ta_3_O_3.66_N_5.34_ phase viewed along the [100] zone axis. Yellow arrows point to the more intense Ta–O/N planes. The inset shows a sketch of the Eu_3_Ta_3_O_3.66_N_5.34_ triple perovskite structure along the [100] zone axis. (c) Two unit-cell-averaged intensity profiles along the direction of the orange arrow are shown in (b). Ta, Eu, O, and N atoms are represented with blue, green/pink, red, and blue circles, respectively.

Figure 8a shows a high-resolution *Z*-contrast image of a Eu_3_Ta_3_O_3.66_N_5.34_ grain viewed along the [100] zone axis. The Fourier Transform (FT) of the image clearly shows the superstructure peaks of the triple cell (indicated by a red bracket). Figure 8b displays a higher-resolution *Z*-contrast image with a magnified view of the superstructure. Notice that every three planes of Ta one is more intense, which allows us to identify and pinpoint the triple perovskite (see yellow arrows in Figure 8b and the intensity profile along the *c*-axis shown in Figure 8c). This is due to the fact that this compound contains two types of Ta–O/N planes (see Figure 6), one with the anions perfectly aligned with Ta cations (Ta2 positions) and another with the anions slightly above or below the Ta plane (Ta1 sites), ensuing slightly dimmer Ta atomic columns compared with the former ones.

### Magnetic Properties

**Figure 9:**
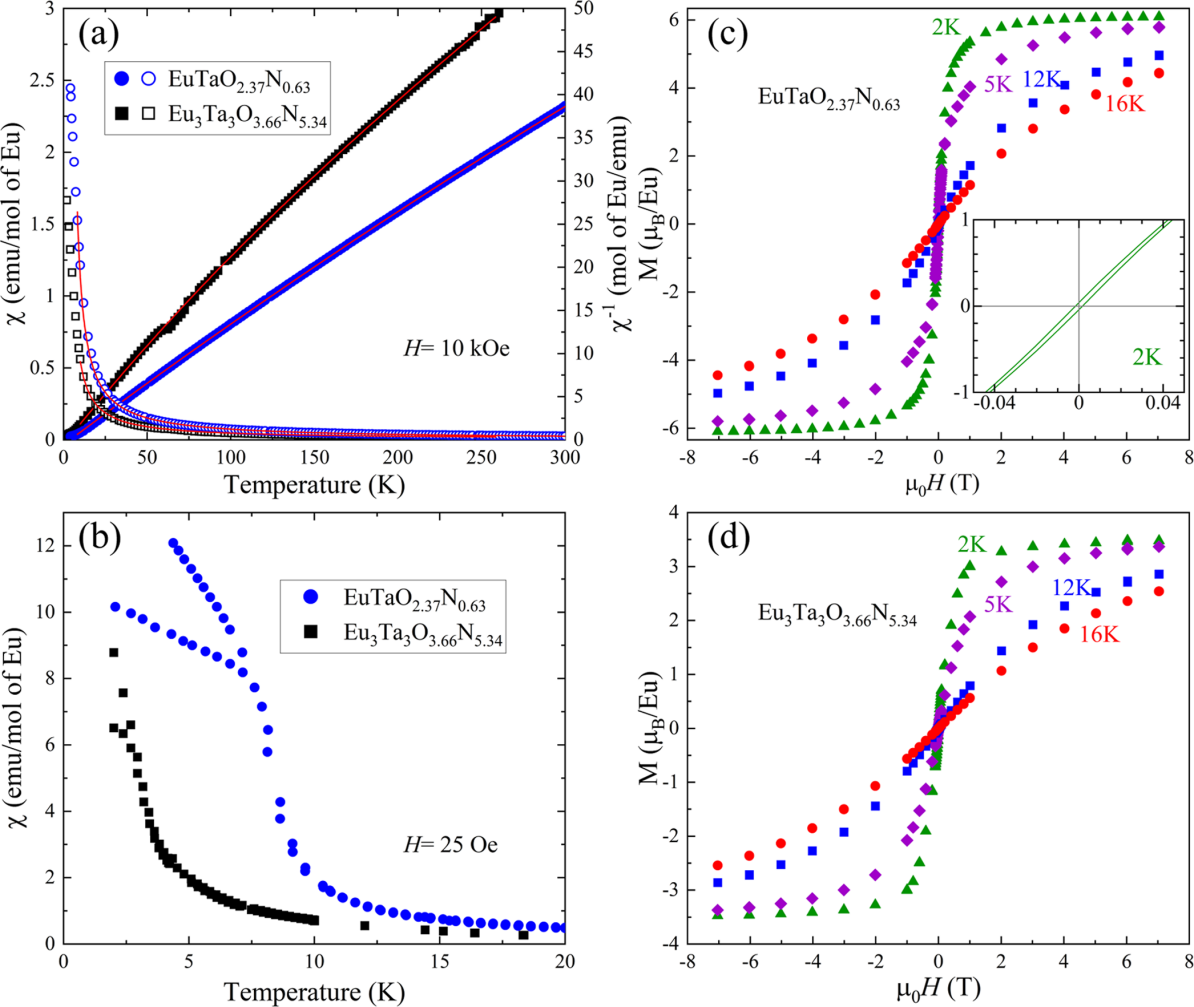
(a) Temperature dependence of the magnetic susceptibility recorded at 10 kOe (left axis) and the inverse susceptibility (right axis) of EuTaO_2.37_N_0.63_ and Eu_3_Ta_3_O_3.66_N_5.34_ together with the fitted values according to eqs 3 and 4, respectively. (b) Temperature dependence of the magnetic susceptibility recorded at a low magnetic field (25 Oe) after zero-field and field-cooling (ZFC-FC) for the same compounds. The corresponding magnetization loops collected between 2 and 16 K are shown in (c,d). Inset in (c) is a zoom of the magnetization loop at 2 K in the low field (<400 Oe) region.

In Figure 9a–d, we summarize the magnetic properties of EuTaO_2.37_N_0.63_ (phase I) and Eu_3_Ta_3_O_3.66_N_5.34_ (phase II). As previously stated, according to the stoichiometric ratios, the charge balance is expected to be (I) Eu^2+^Ta_0.63_^5+^Ta_0.37_^4+^O_2.37_N_0.63_ and (II) Eu_2.34_^3+^Eu_0.66_^2+^Ta_3_^5+^O_3.66_N_5.34_.

The temperature-dependent magnetic susceptibility χ(T) of phase I is expected to display Curie–Weiss (CW) behavior governed by the presence of Eu^2+^ (4f^7^ (^8^S)) ions having localized *S* = 7/2 spin. The presence of 5d^1^ electrons (Ta^4+^ ions) in a partially occupied broadband is expected to produce a marginal temperature-independent Pauli paramagnetism that will add to any diamagnetic contribution. Accordingly, χ(T) is given by
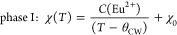
3where *C*(Eu^2+^) is the corresponding Curie constant and θ_CW_ is the extrapolated Curie temperature that give a measure of the strength of the magnetic interactions between the spins, eventually ordered at low temperature. χ_0_ contains temperature-independent paramagnetic and diamagnetic susceptibilities. If the 5d^1^ electrons are spin-polarized by the magnetic moments of Eu^2+^ ions, a departure from the χ(*T*) dependence described by eq 3 is expected. This has been observed for instance in Sr_2_FeMoO_6_,^32^ where localized moments of 3d-Fe^2+/3+^ ions induce a spin polarization in the conduction band (4d-Mo^4+^).

For Eu_3_Ta_3_O_3.66_N_5.34_ (phase II), the presence of localized moments at Eu^2+^ ions should produce a CW contribution to χ(T) as described above, of relative weight “*n*_Eu^2+^_” combined with the temperature-dependent van Vleck contribution of the magnetic moment of Eu^3+^.^33,34^ Notice that although Eu^3+^ in its ground state is nonmagnetic (^7^F_0_), thermal excitation to higher lying states (for instance the first one (^7^F_1_) is only at about 46 meV^33^ and shall produce a temperature-dependent magnetic susceptibility that will add to the Eu^2+^ contribution, of weight (1 – *n*_Eu^2+^_), and to any diamagnetic contribution). Accordingly, the magnetic susceptibility per Eu ion can be expressed as
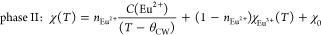
4The magnetic susceptibility recorded at 10 kOe of these compounds displays roughly high-temperature CW behavior (Figure 9a, right axis), where some curvature can be readily appreciated more apparently for phase II than for phase I, as expected from eqs 3 and 4.

Equation 3 and 4 have been used to fit the data for EuTaO_2.37_N_0.63_ and Eu_3_Ta_3_O_3.66_N_5.34_, respectively. The van Vleck contribution to the susceptibility of Eu^3+^ was computed using an excitation energy of 46 meV as given in ref (33). Continuous lines through the data in Figure 9a are the results of fitting eqs 3 and 4 to the experimental χ(*T*) curves and the corresponding fitted parameters are listed in Table 2.

**Table 2:** Parameters Obtained from Fittings to Magnetic Susceptibility Data of EuTaO_2.37_N_0.63_ Using Equation 3, and for Eu_3_Ta_3_O_3.66_N_5.34_ Using Equation 4 by Fixing  and

	*n*_Eu^2+^_	μ_eff_(μ_B_)	θ_CW_ (K)	χ_0_ (emu/mol)
EuTaO_2.37_N_0.63_	1	7.44	4.7	2.5 × 10^–3^
Eu_3_Ta_3_O_3.66_N_5.34_	0.49	7.94 (fixed)	2.4	1.7 × 10^–3^

Data in Table 2 reflect the dominating presence of Eu^2+^ ions in EuTaO_2.37_N_0.63_. The extracted effective moment (μ_eff_ ≈ 7.44 μ_B_/f.u.) compares well with the expected one (7.94 μ_B_/f.u.) for Eu^2+^ (*S* = 7/2) ions. The extracted θ_CW_ (≈4.7 K) implies that ferromagnetic order should be expected at around this temperature. Indeed, the magnetization data recorded under 25 Oe after zero-field and field-cooling processes (ZFC and FC), shown in Figure 9b, clearly display a hysteretic behavior, developing around 7.5 K. The corresponding field-dependent magnetization loops measured at various temperatures are shown in Figure 9c. The shape of the *M*(*H*) curves is consistent with a ferromagnetic ordering, with a saturation magnetization of about 6 μ_B_, closely approaching the nominal 7 μ_B_ contribution from Eu^2+^ expected for Eu^2+^Ta_0.63_^5+^Ta_0.37_^4+^O_2.37_N_0.63_, and coinciding with the effective moment extracted from susceptibility curves in Figure 9a. Detailed inspection of the low field range in the *M*(*H*) data taken at 2 K (Figure 9c (inset)) shows the presence of a minor hysteresis, again consistent with the ferromagnetic character of the sample.

The magnetic data of the nitrogen-richer Eu_3_Ta_3_O_3.66_N_5.34_ sample reveals that the effective magnetic moment per Eu ion is largely suppressed and the Curie–Weiss temperature drops by about 50% down to ≈2.4 K. These observations are consistent with the larger fraction of the nonmagnetic Eu^3+^ ions as inferred from *n*_Eu^3+^_ ≈ 0.51 (Table 2). The corresponding ZFC-FC data (Figure 9b) confirm that ferromagnetic order develops only at lower temperatures (≈3 K). The *M*(*H*) curves (Figure 9d) consistently reflect a dramatic reduction of the saturation magnetization (≈3.5 μ_B_). The relative fraction of Eu^2+^ ions in the phase II sample deduced from susceptibility data in Figure 9a (*n*_Eu^2+^_ ≈ 0.49) is larger than expected from chemical analysis (*n*_Eu^2+^_ ≈ 0.22). This difference could originate from the possible existence of anion vacancies, which have not been considered and would increase the proportion of Eu^2+^, as well as from the extreme simplification of eq 4. For instance, a concentration of oxygen vacancies of 4.7% (0.42 atoms) would lead to *n*_Eu^2+^_ = 0.5, involving an increase of the fraction of this cation in both A1 and A2 sites of the triple perovskite structure.

All in all, the magnetization data in Figure 9 allow us to conclude that by increasing the N/O ratio in europium tantalum perovskite oxynitrides, the magnetization reduces and the ferromagnetic ordering temperature lowers by the increasing contribution of the nonmagnetic Eu^3+^ in the structure, that dilutes magnetic interaction among Eu^2+^ ions.

## Conclusions

A new high-temperature solid-state synthesis approach under N_2_ or N_2_/H_2_ gas at 1200 °C is used to obtain europium perovskite tantalum oxynitrides with a large range of nitrogen contents, starting with mixtures of Eu_2_O_3_ and TaON or Eu_2_O_3_, EuN, and Ta_3_N_5_. EuTaO_2.37_N_0.63_ prepared from Eu_2_O_3_ and TaON under N_2_/H_2_ shows a simple cubic *Pm*3̅*m* perovskite structure whereas the new, highly nitrided compound Eu_3_Ta_3_O_3.66_N_5.34_ is prepared from Eu_2_O_3_, EuN, and Ta_3_N_5_. Eu_3_Ta_3_O_3.66_N_5.34_ with formal stoichiometry Eu_2.34_^3+^Eu_0.66_^2+^Ta_3_O_3.66_N_5.34_ is a mixed-valence Eu^2+^/Eu^3+^ compound with long-range order of europium ions in two A sites with different average charge and ratio 2:1, occupied preferentially by Eu^3+^ and Eu^2+^ respectively, that generate well-differentiated coordination environments. This order leads to a triple perovskite structure crystallizing in the *P*4/*mmm* space group with parameters *a* = a_0_, *c* = 3*a*_0_, where *a*_0_ is the parameter of the cubic perovskite subcell. The new perovskite is ferromagnetic with *T*_c_ ≈ 3 K and saturation magnetization of ≈3 μ_B_, which are lower than for EuTaO_2.37_N_0.63_ (*T*_c_ ≈ 8 K, *M*_s_ ≈ 6 μ_B_) because of the presence of Eu^3+^, which has a nonmagnetic ground state and dilutes the magnetic interactions between the Eu^2+^ cations. These findings increase the diversity of crystal structures in the field of perovskite oxynitrides and demonstrate that the synthesis from mixtures of binary nitrides and oxides is very effective in tuning their nitriding degree when cations in different oxidation states can be present by controlling the N/O ratio in the reactants. The same synthetic approach could be extended to other perovskite oxynitrides, potentially leading to new structures and physical properties by expanding the accessed anion compositions of the compounds prepared by ammonolysis.

## References

[ref1] Fuertes A. Nitride Tuning of Transition Metal Perovskites. APL Mater. 2020, 8, 020903 10.1063/1.5140056.

[ref2] Yang M.; Oró-Solé J.; Rodgers J. A.; Jorge A. B.; Fuertes A.; Attfield J. P. Anion Order in Perovskite Oxynitrides. Nat. Chem. 2011, 3, 47–52. 10.1038/nchem.908. 21160517

[ref3] Porter S. H.; Huang Z.; Woodward P. M. Study of Anion Order/Disorder in RTaN_2_O (R = La, Ce, Pr) Perovskite Nitride Oxides. Cryst. Growth Des. 2014, 14, 117–125. 10.1021/cg401230a.

[ref4] Attfield J. P. Principles and Applications of Anion Order in Solid Oxynitrides. Cryst. Growth Des. 2013, 13, 4623–4629. 10.1021/cg4011168.

[ref5] Ceravola R.; Oró-Solé J.; Black A. P.; Ritter C.; Puente Orench I.; Mata I.; Molins E.; Frontera C.; Fuertes A. Topochemical Synthesis of Cation Ordered Double Perovskite Oxynitrides. Dalton Trans. 2017, 46, 5128–52131. 10.1039/C7DT00800G. 28345706

[ref6] Ceravola R.; Frontera C.; Oró-Solé J.; Black A. P.; Ritter C.; Mata I.; Molins E.; Fontcuberta J.; Fuertes A. Topochemical Nitridation of Sr_2_FeMoO_6_. Chem. Commun. 2019, 55, 3105–3108. 10.1039/C8CC09845J. 30789159

[ref7] Ishida K.; Tassel C.; Watabe D.; Takatsu H.; Brown C. M.; Nilsen G. J.; Kageyama H. Spin Frustration in Double Perovskite Oxides and Oxynitrides: Enhanced Frustration in La_2_MnTaO_5_N with a Large Octahedral Rotation. Inorg. Chem. 2021, 60 (2021), 8252–8258. 10.1021/acs.inorgchem.1c00927. 34029076 PMC10494547

[ref8] Ruddlesden S. N.; Popper P. New Compounds of the K_2_NiF_4_ type. Acta Crystallogr. 1957, 10, 538–539. 10.1107/S0365110X57001929.

[ref9] Ruddlesden S. N.; Popper P. The Compound Sr_3_Ti_2_O_7_ and its Structure. Acta Crystallogr. 1958, 11, 54–55. 10.1107/S0365110X58000128.

[ref10] Tobías G.; Oró-Solé J.; Beltrán-Porter D.; Fuertes A. New Family of Ruddlesden-Popper Strontium Niobium Oxynitrides: (SrO)(SrNbO_2-x_N)_n_ (n = 1, 2). Inorg. Chem. 2001, 40, 6867–6869. 10.1021/ic015566i. 11754265

[ref11] Pors F.; Marchand R.; Laurent Y. Nouveaux Oxynitrures A_2_TaO_3_N (A = Alcalinoterreux) de type structural K_2_NiF_4_. Ann. Chim. 1991, 16, 547–551.

[ref12] Clarke S. J.; Hardstone K. A.; Michie C. W.; Rosseinsky M. J. High-Temperature Synthesis and Structures of Perovskite and n = 1 Ruddlesden–Popper Tantalum Oxynitrides. Chem. Mater. 2002, 14, 2664–2669. 10.1021/cm011738y.

[ref13] Cordes N.; Nentwig M.; Eisenburger L.; Oeckler O.; Schnick W. Ammonothermal Synthesis of the Mixed-Valence Nitrogen-Rich Europium Tantalum Ruddlesden-Popper Phase Eu^II^Eu_2_^III^Ta_2_N_4_O_3_. Eur. J. Inorg. Chem. 2019, 2019, 2304–2311. 10.1002/ejic.201900245.

[ref14] Marchand R. Oxynitrures à Structure K_2_NiF_4_. Les composés Ln_2_AlO_3_N (Ln= La, Nd, Sm). C.R. Acad. Sci. 1976, 282, 329–331.

[ref15] Dion M.; Ganne M.; Tournoux M. Nouvelle Famille du phases M^I^M_2_^I II^ Nb_3_O_10_. Mater. Res. Bull. 1981, 16, 1429–1435. 10.1016/0025-5408(81)90063-5.

[ref16] Jacobson A. J.; Johnson J. W.; Lewandowski J. T. Interlayer Chemistry between Thick Transition-Metal Oxide Layers: Synthesis and Intercalation Reactions of K[Ca_2_Na_n-3_Nb_n_O_3n+1_] (3 ≤ n ≤ 7). Inorg. Chem. 1985, 24, 3727–3729. 10.1021/ic00217a006.

[ref17] Schottenfeld J. A.; Benesi A. J.; Stephens P. W.; Chen G.; Eklund P. C.; Mallouk T. E. Structural Analysis and Characterization of Layer Perovskite Oxynitrides made from Dion–Jacobson Oxide Precursors. J. Solid State Chem. 2005, 178, 2313–2321. 10.1016/j.jssc.2005.05.012.

[ref18] Oshima T.; Ichibha T.; Oqmhula K.; Hibino K.; Mogi H.; Yamashita S.; Fujii K.; Miseki Y.; Hongo K.; Lu D.; Maezono R.; Sayama K.; Yashima M.; Kimoto K.; Kato H.; Kakihana M.; Kageyama H.; Maeda K. Two-Dimensional Perovskite Oxynitride K_2_LaTa_2_O_6_N with an H^+^/K^+^ Exchangeability in Aqueous Solution Forming a Stable Photocatalyst for Visible-Light H_2_ Evolution. Angew. Chem., Int. Ed. 2020, 59, 9736–9743. 10.1002/anie.202002534. 32134159

[ref19] Oró-Solé J.; Fina I.; Frontera C.; Gàzquez J.; Ritter C.; Cunquero M.; Loza-Alvarez P.; Conejeros S.; Alemany P.; Canadell E.; Fontcuberta J.; Fuertes A. Engineering Polar Oxynitrides: Hexagonal Perovskite BaWON_2_. Angew. Chem., Int. Ed. 2020, 59, 18395–18399. 10.1002/anie.202006519. 32649790

[ref20] Jorge A. B.; Oró-Solé J.; Bea A. M.; Mufti N.; M Palstra T. T.; Rodgers J. A.; Attfield J. P.; Fuertes A. Large Coupled Magnetoresponses in EuNbO_2_N. J. Am. Chem. Soc. 2008, 130, 12572–12573. 10.1021/ja804139g. 18759396

[ref21] Yang M.; Oró-Solé J.; Kusmartseva A.; Fuertes A.; Attfield J. P. Electronic Tuning of Two Metals and Colossal Magnetoresistances in EuWO_1+x_N_2–x_ Perovskites. J. Am. Chem. Soc. 2010, 132, 4822–4829. 10.1021/ja910745b. 20218601

[ref22] Mikita R.; Aharen T.; Yamamoto T.; Takeiri F.; Ya T.; Yoshimune W.; Fujita K.; Yoshida S.; Tanaka K.; Batuk D.; Abakumov A. M.; Brown C. M.; Kobayashi Y.; Kageyama H. Topochemical Nitridation with Anion Vacancy-Assisted N^3–^/O^2–^ Exchange. J. Am. Chem. Soc. 2016, 138, 3211–3217. 10.1021/jacs.6b00088. 26855196

[ref23] Marchand R.; Pors F.; Laurent Y. Nouvelles Perovskites Oxynitrures de Stoechiometrie ABO_2_N (A= Lanthanide, B = Ti) et ABON_2_ (A= Lanthanide, B = Ta ou Nb). Ann. Chim. 1991, 16, 553–560.

[ref24] Oró-Solé J.; Frontera C.; Black A. P.; Castets A.; Velásquez-Méndez K. L.; Fontcuberta J.; Fuertes A. Structural, Magnetic and Electronic Properties of EuTi_0.5_W_0.5_O_3-x_N_x_ Perovskite Oxynitrides. J. Solid State Chem. 2020, 286, 121274 10.1016/j.jssc.2020.121274.

[ref25] Brauer G.; Weidlein J.; Strähle J. Uber das Tantalnitrid Ta_3_N_5_ und das Tantaloxidnitrid TaON. Z. Anorg. Allg. Chem. 1966, 348, 298–308. 10.1002/zaac.19663480511.

[ref26] Fauth F.; Peral I.; Popescu C.; Knapp M. The New Material Science Powder Diffraction Beamline at ALBA Synchrotron. Powder Diffr. 2013, 28, S360–S370. 10.1017/S0885715613000900.

[ref27] Rodríguez-Carvajal J. Recent Advances in Magnetic Structure Determination by Neutron Powder Diffraction. Phys. B 1993, 192, 55–69. 10.1016/0921-4526(93)90108-I.

[ref28] Castets A.; Fina I.; Guarín J. R.; Oró-Solé J.; Frontera C.; Ritter C.; Fontcuberta J.; Fuertes A. High-Temperature Synthesis and Dielectric Properties of LaTaON_2_. Inorg. Chem. 2021, 60, 16484–16491. 10.1021/acs.inorgchem.1c02325. 34623795

[ref29] Sakata T.; Yoshiyuki R.; Okada R.; Urushidani S.; Tarutani N.; Katagiri K.; Inumaru K.; Koyama K.; Masubuchi Y. Environmentally Benign Synthesis and Color Tuning of Strontium–Tantalum Perovskite Oxynitride and its Solid Solutions. Inorg. Chem. 2021, 60, 4852–4859. 10.1021/acs.inorgchem.0c03758. 33631931

[ref30] Bubeck C.; Widenmeyer M.; Richter G.; Coduri M.; Goering E.; Yoon S.; Weidenkaff A. Tailoring of an Unusual Oxidation State in a Lanthanum Tantalum(IV) Oxynitride Via Precursor Microstructure Design. Comm. Chem. 2019, 2, 134 10.1038/s42004-019-0237-x.

[ref31] Shannon R. D. Revised Effective Ionic Radii and Systematic Studies of Interatomic Distances in Halides and Chalcogenides. Acta Crystallogr., Sect. A: Cryst. Phys., Diffr., Theor. Gen. Crystallogr. 1976, 32, 751–767. 10.1107/S0567739476001551.

[ref32] Serrate D.; De Teresa J. M.; Ibarra M. R. Double Perovskites with Ferromagnetism above Room Temperature. J. Phys.: Condens. Matter 2007, 9, 023201 10.1088/0953-8984/19/2/023201.

[ref33] Andruh M.; Bakalbassis E.; Kahn O.; Trombe J. C.; Porcher P. Structure, Spectroscopic and Magnetic Properties of Rare Earth Metal(III) Derivatives with the 2-formyl-4-methyl-6-(N-(2-pyridylethyl)formimidoyl)phenol Ligand. Inorg. Chem. 1993, 32, 1616–1622. 10.1021/ic00061a017.

[ref34] Zong Y.; Fujita K.; Akamatsu H.; Murai S.; Tanaka K. Antiferromagnetism of Perovskite EuZrO_3_. J. Solid State Chem. 2010, 183, 168–172. 10.1016/j.jssc.2009.10.014.

